# Editorial: Secondary metabolism: an unlimited foundation for synthetic biology, volume II

**DOI:** 10.3389/fmicb.2023.1200928

**Published:** 2023-05-17

**Authors:** Ana Lúcia Leitão, Francisco J. Enguita

**Affiliations:** ^1^MEtRICs, Department of Chemistry, NOVA School of Science and Technology, FCT NOVA, Universidade NOVA de Lisboa, Caparica, Portugal; ^2^Faculdade de Medicina, Instituto de Medicina Molecular João Lobo Antunes, Universidade de Lisboa, Lisbon, Portugal

**Keywords:** secondary metabolism, synthetic biology, microorganisms, biotechnology, biological modules

Synthetic Biology relies on the concept that biological systems are organized into modular components, that can be easily assembled, tested, and modified to generate biological machineries (Bhattacharya et al., 2022). The biological modules can be genetic circuits, enzymes, metabolic pathways, or even entire genomes, that can be rationally combined to achieve a desired output (Ghosh, 2022). The successful implementation of the rationale of Synthetic Biology requires a deep understanding of the rules governing the function of all the biological modules (Shih and Chen, 2022). We compiled five articles describing different metabolic modules directly or indirectly involved in secondary metabolism, that could be employed as building blocks of synthetic biology concepts with applications in different fields including the environmental restoration of diversity, human nutrition, and health.

Synthetic biology explores the directed metabolic alterations to produce high-value products transforming microorganisms into chemical factories. Lignocellulosic biomass has been suggested as an alternative to fossil fuels being a promising renewable resource from an economic and environmental perspective (Pfleger and Takors, 2023). D-xylose is the predominant pentose of lignocellulosic biomass and its utilization is a pre-requisite for producing biofuels sustainably. One critical processing step required to produce biofuels hinges upon microorganism's metabolism to reach high productivity through an efficient carbon flux. A manuscript by Cheng et al. published in this Research Topic, described an epigenetic link that connects xylose metabolization with amino acid biosynthesis in strains of *Saccharomyces cerevisiae*. Targeted deletion of the NGG1, a member of the chromatin-modifying histone acetyltransferase complex, resulted in an increase of xylose metabolism linked to a down-regulation of the transcription of genes related to mitochondrial function, NADH generation, amino acid and ATP biosynthesis, as well as TCA cycle (Cheng et al.).

Another paper by Fischer et al. studied a very interesting fungal strain, *Pyronema domesticum*, isolated from the burned soil after the catastrophic 2013 Rim Fire event (CA, United States). This strain showed an improved ability to metabolize pyrolyzed organic matter (PyOM), a relative recalcitrant subproduct resulting from the combustion of vegetal organic matter during wildfires. Using high resolution transcriptomic analysis, the authors of the manuscript investigated the response of *P. domesticum* to PyOM observing an induction pattern of genes related to stress tolerance, in addition to metabolism and mineralization of aromatic compounds. The mineralization of PyOM by the dominant early-successional fungus *P. domesticum* is likely to have broad impacts on post-fire succession and recovery in soil microbial communities (Fischer et al.).

Additionally, environmental stress due to abiotic factors such as osmolarity impose considerable energetic and growth constrains on bacterial cells in their growing habitats (Gregory and Boyd, 2021). In this context L-proline is an essential member of the compatible solute family, widely used by both plants and microorganisms as an osmoprotectant (Per et al., 2017). An article by Stecker et al., describes the functional characterization of the L-proline biosynthetic route by osmotolerant strains of *Bacillus subtilis*. A genomic analysis of osmotolerant strains determined the presence of mutations affecting either the AhrC transcriptional regulator or its operator positioned in front of the *argCJBD-carAB-argF* L-ornithine/L-citrulline/L-arginine biosynthetic operon. These mutations, together with regulatory mutations affecting *rocR-rocDEF* expression, were responsible for the repurposing of three different routes: L-arginine biosynthesis, RocD-dependent degradation for L-ornithine, and the last step in L-proline biosynthesis. The authors concluded that these genetic adaptations are a demonstration of the genetic plasticity and metabolic flexibility of *B. subtilis* to counteract the changing environmental conditions (Stecker et al.).

Two additional papers reported studies on secondary metabolites with potential interest in the areas of human health, specifically nutrition and therapeutics. In the nutritional field, long chain polyunsaturated omega-3 fatty acids such as docosahexaenoic acid (DHA) are essential fatty acids for human health with fish oil being its current main source. Due to DHA high demand for pharmaceutical and nutraceutical purposes, microorganisms have been described as an emerging alternative source of this omega-3 fatty acid. Song et al. studied the effects of cold stress accompanied with staged-temperature control on the fatty acid metabolism of *Aurantiochytrium* sp., a heterotrophic unicellular marine thraustochytrid able to produce DHA. The authors analyzed the metabolic response of the microorganism when submitted to cold stress (15 and 5°C), quantifying the physiological responses (morphology, growth, fatty acid profiling) and the expression of genes related with fatty acid biosynthesis. They concluded that the treatment of the cells at 5°C increases DHA biosynthesis. Moreover, this metabolic increment was accompanied by a transcriptional induction of the metabolic enzymes involved in fatty acid biosynthesis, determined by qPCR (Song et al.).

Antibiotics are one of the most relevant products of secondary metabolism from microorganisms, showing a wide chemical diversity and action mechanisms. The production of an antibiotic is tightly regulated, and the study of the molecular details of this regulation has become an essential topic to develop improved strains with enhanced biosynthetic potential (Zhang et al., 2022). The work by Zhang et al. describes the molecular basis for the transcriptional regulation of the biosynthesis of the antibiotics 2,4-diacetyphloroglucinol (2,4-DAPG) and pyoluteorin by *Pseudomonas protegens* FD6. In this bacterial strain, the sigma factor RpoS is an essential regulator of cell cycle. RpoS negative mutants showed an impairment of biofilm formation, swimming motility, swarming motility, and resistance to chemical stress, but an increase in the antibiotic biosynthesis. The authors concluded that RpoS negatively controlled 2,4-DAPG biosynthesis and transcription of the 2,4-DAPG operon *phlACBD* by directly interacting with *phlG* and *phlA* promoters. Moreover, RpoS also inhibited the production of pyoluteorin by a transcriptional repression mechanism of its biosynthetic operon *pltLABCDEFG* exerted by a direct binding to the promoter regions of *pltR, pltL* and *pltF* genes (Zhang et al.).

In summary, despite the multiple biological functions that secondary metabolites could have, they are very relevant to understand the biological response to stress conditions, opening a new avenue of opportunities for synthetic biology applications.

## Author contributions

AL and FE: conceptualization, idea, writing, organization, and writing the manuscript. All authors contributed to the article and approved the submitted version.
